# Comparative Assessment of Crestal Bone Loss by Flapless and Flap Technique for Implant Placement: A Prospective Study

**DOI:** 10.7759/cureus.38598

**Published:** 2023-05-05

**Authors:** Archana A, Rupamalini SN, Surya Dahiya, Prashant Babaji, Atul Anand Bajoria, Gangadhar K

**Affiliations:** 1 Oral Pathology, Autonomous State Medical College, Etah, IND; 2 Periodontology, Mathrusri Ramabai Ambedkar Dental College & Hospital, Bangalore, IND; 3 Conservative Dentistry and Endodontics, Maharishi Markandeshwar College of Dental Sciences & Research, Mullana, IND; 4 Pedodontics and Preventive Dentistry, Sharavathi Dental College & Hospital, Shimoga, IND; 5 Oral Medicine and Radiology, Kalinga Institute of Dental science, Bhubaneswar, IND; 6 Oral and Maxillofacial Surgery, P.M.Nadagouda Memorial Dental College & Hospital, Bagalkot, IND

**Keywords:** flap, bone loss, crestal, flap-less, implant

## Abstract

Background: The use of flapless surgery for placing dental implants is extremely popular due to better radiological tools and the availability of software that help in the planning of dental implants.

Objective: The present study was done to assess crestal bone loss by using flapless and flap techniques for placing implants.

Methods: A total of 50 subjects who satisfied the inclusion criteria were selected for this study. Selected patients were then divided equally into two study groups i.e., those who are and those who are not undergoing flap surgery. Statistical analysis was done using the Mann-Whitney U test.

Results: Statistically considerable P values were obtained. Bone loss was lesser with the flapless technique.

Conclusion: Flapless implant placement demonstrated less crestal bone loss compared to flap surgery.

## Introduction

Osseointegration is essential for successful implant placement and is based on the interface forming between an implant and the bone. It is a biological consequence of a well-performed surgical procedure, which in some instances is not achieved and can lead to implant failure. Osseointegration in dental implantation is typically done by using a flap-based approach that involves reflection of soft tissue flap and also necessitates suturing following the placement of dental implants [1]. In the conventional procedure for implant placement, the flap is generally raised for better visualization of the operated area. Elevation of the flap helps in the proper identification of the anatomical landmark, and the available bone is assessed to facilitate implant placement. However, this approach has been linked with a certain amount of morbidity as well as discomfort. The main problem is the loss of alveolar crestal bone due to a decrease in supra-periosteal vascular supply. In addition, other related changes may include postoperative loss of blood and/or hemorrhage, and pain alongside discomfort for a patient [2,3]. 

Hence, to circumvent these main drawbacks, a flapless approach has been developed wherein a smaller bit of soft tissue overlying the edentulous crestal ridge undergoes surgical removal which is sufficient for the placement of an implant. There is no requirement for sutures since no reflection of the soft tissue flap is done, thereby reducing the time of surgery. Also, soft as well as hard tissue levels are maintained which can significantly reduce postoperative swelling as well as discomfort. 

The use of the flapless technique for the placement of an implant has shown a positive outcome in the preservation of the height of alveolar crestal bone and the process of osseointegration compared to the technique utilizing the flap [4]. However, there is a lack of sufficient documentation related to studies that have been carried out among humans. This study aims to evaluate and compare changes in the height of crestal bone surrounding implants that have been inserted using both flapless and flap surgeries. Before the study, a null hypothesis was made that a flapless surgical procedure causes less reduction of alveolar bone compared to flap surgery.

## Materials and methods

Study design

The present study is a prospective, cross-sectional, single-center study performed by a single operator. Ethical clearance was obtained from the Institutional Review Board of P.M.Nadagouda Memorial Dental College & Hospital (approval no. PMNMCDCH/ 2475/2019-20). Informed consent was obtained from all the participants. A total of 50 patients who wanted to replace missing teeth were included for the insertion of implants in the posterior mandibular region by flap, and flapless techniques. A total of 50 tapered threaded implants, (Vision™ Hi‑Tec implants, Lifecare Devices Private Ltd., New Delhi, India) were used in both the groups to avoid bias. Subjects were divided into two groups: Group I had 25 subjects who were treated with flap surgery (n=25), and Group II included patients who had flapless surgery (n=25). Implant placement was guided through radiographic and clinical evaluation of the alveolar crest. In both groups, the selected implants were inserted within osteotomy sites with implant flat form placed at the sub crestal level.

Inclusion and exclusion criteria

Inclusion criteria were as follows: subjects above 18 years of age; a sufficient width of bone measuring a minimum of 4.5 mm near the crest (the distance measured between the apical ends of the first thread of the implant to the most coronal point of the interproximal crestal bone at the mesial and distal aspect of the implant) and without any undercuts of greater than 15 degrees when measured on a radiograph; the presence of keratinized soft tissue measuring a minimum of 5 mm with the aid of William’s periodontal probe. Participants with any type of comorbidities and individual risk factors were evaluated and not included in the study. Cases with type II bone type in the mandible were chosen in both groups.

Pre-surgery assessment

Complete patient histories were obtained before the selection for implant placement procedure. A complete evaluation of all vital signs and a hemogram were done to evaluate patient fitness before proceeding with implant insertion. This was followed by thorough oral prophylaxis. Preoperative intraoral periapical radiographs (IOPAs) and orthopantomograms (OPGs) were taken for obtaining the required information on the available bone and the distance between vital anatomical structures such as the inferior alveolar (mandibular) canal and/or mental foramen from the site of implant placement. Any source of infection present in the mouth was treated before the implant procedure.

Before implant placement, information about risk factor which affects implant success was evaluated, such as systemic (diabetes, cardiac issues) and local factors (smoking, periodontal condition, cause of tooth loss, edentulous duration, implant, and bone condition) and belonging to an older age group.

Surgical procedure

Implant Insertion Using Flap Surgery

**Figure 1:**
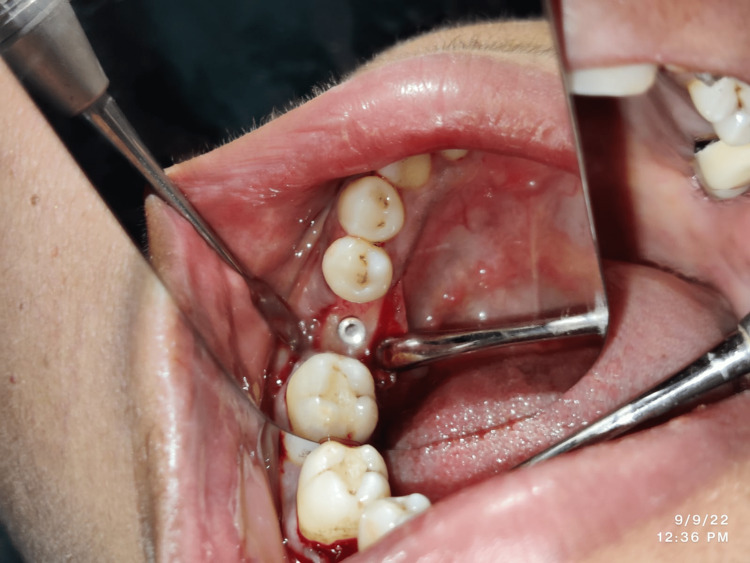
Flap reflection during implant placement in the (conventional) flap surgery group

The local anesthetic agent was administered at the selected edentulous site for implant insertion by injecting 2% lignocaine with adrenalin in a ratio of 1:100,000. The placement of the implant by use of conventional flap surgery involved a crestal surgical incision for reflection of full-thickness flap to enable exposure of the site (Figure 1).

**Figure 2:**
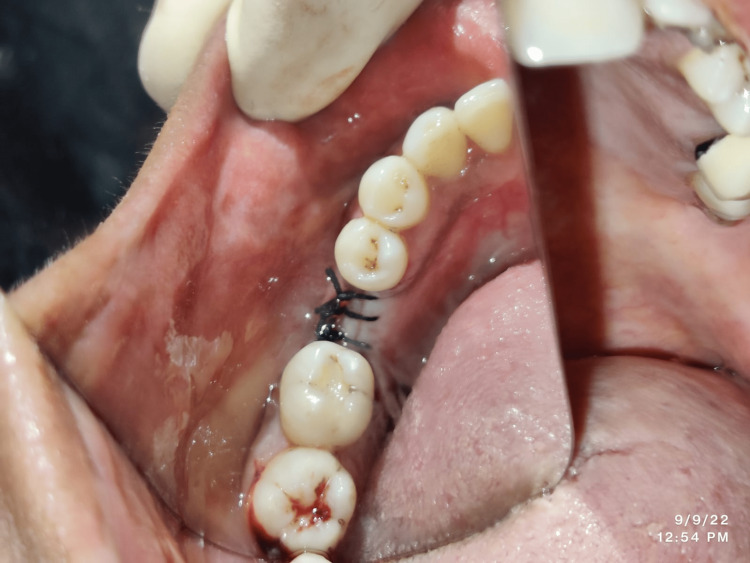
Suturing after implant placement in the flap surgery group

A surgical stent was placed on the alveolar crest to mark the site of implant insertion. The implant site was then marked for creating bleeding points and for performing the initial step of the osteotomy. A pilot drill was used with a subsequent increase in drill diameter. A final drill was then used till the pre-decided depth to create a site of osteotomy with specific dimensions for each of the patients. The selected implants were inserted within these osteotomy sites. This was followed by the placement of healing abutments onto the implants immediately after their placement and to close the opened site. Then, thorough irrigation was done, and flap closure with tightly placed using non-resorbable 3-0 silk sutures (Figure 2). All patients in Group I were then prescribed a course of antibiotics along with analgesics for a postoperative period of one week.

Implant Insertion Using Flapless Surgery

**Figure 3:**
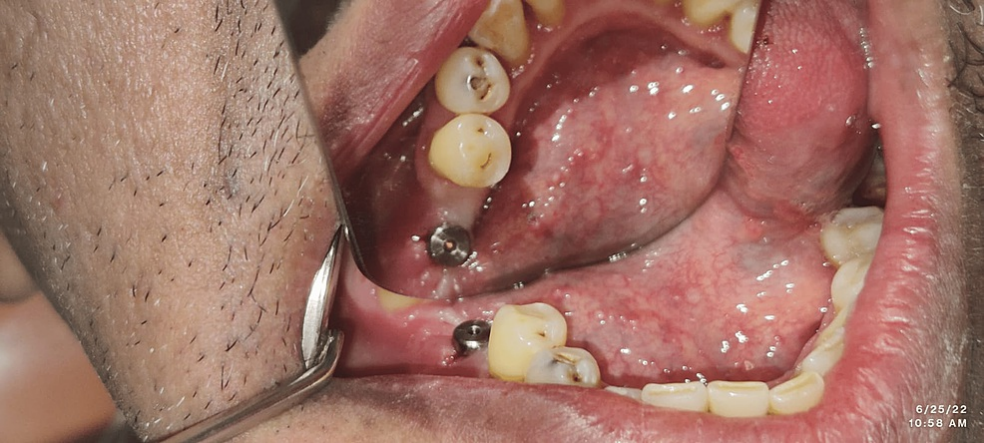
Implant placement in the flapless surgery group

The routine protocol for preoperative procedures in Group II was similar to the patients in Group 1. Patients in Group II had no elevation of the flap. The reference template was used in implant site preparation which acts as a key guide for the flapless technique. For the flapless technique, a punch of tissue was removed from the intended implant placement site, and then sequential drilling was performed to obtain a final osteotomy to receive the implant. A surgical stent was placed within a patient’s oral cavity and the selected site for implant insertion was then marked by a soft tissue punch in each of the patients. Post the removal of the surgical stent, a similar-sized soft tissue punch was employed for the removal of overlying soft tissue at the site of implant placement. Then, a pilot drill was used with a subsequent increase in the sizes of drills. This was followed by final drilling to the adequate depth to create a site for osteotomy. Selected implants were then inserted within these osteotomy sites (Figure 3). 

Placement of Implants & Prosthesis

All implants (in both groups) were finally inserted slightly below the alveolar crestal bone level. This was immediately followed by the screwing of healing abutments. After the placement of the implants by either of these techniques, the implants were then kept for osseointegration that followed early protocols for loading which is two months (for the mandible) [5].

Postoperative instructions involved details on the type of diet and maintenance of oral hygiene. All patients were recalled 24 hours postoperatively for a review, and after one week for assessment of postoperative healing and the removal of sutures in patients of Group I.

Following the placement of an abutment, an impression was made with polyvinyl siloxane impression material via the ‘direct impression’ technique. This impression was then poured into using a die stone for the fabrication of a cast, after which the cutting of the die was done. This was followed by the fabrication of a wax pattern. Then, the metal casting was fabricated by investing as well as casting the wax pattern.

The metal try-in was followed by an appropriate selection of shade. The final prosthesis was then fabricated and tried within a patient’s oral cavity. Occlusion was adjusted and following the final trial, the prosthesis was cemented using type I glass ionomer cement (GIC).

Follow-up

**Figure 4:**
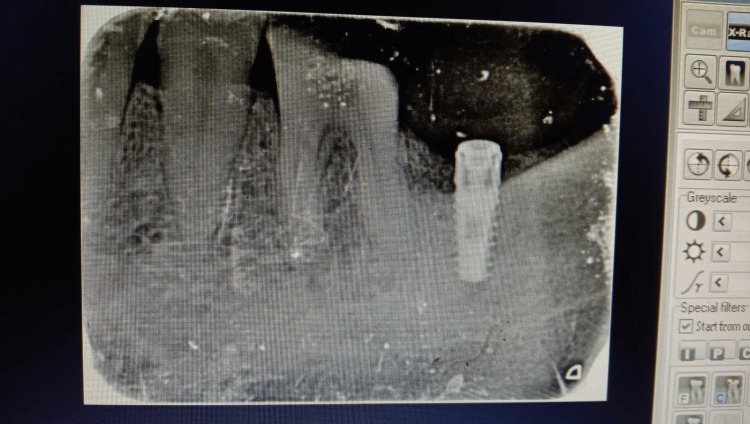
Radiographic postoperative view of implant position

Each patient was recalled for follow-up by radiographical evaluation at six weeks, 12 weeks, and six months following the placement of implants to evaluate changes in crestal bone heights around the implant (Figure 4). 

Standardized intra-oral peri-apical radiographs were made at six weeks, 12 weeks, and six months postoperatively, and were digitized by image analysis software XVa3 (Unicorn Denmart, New Delhi, India). These measurements were done after calibrating the software to avoid any error in the values. A pre-standardized and pre-calibrated length of an implant was used for calibrating digitized images stored in computer software.

Statistical analysis

Obtained results were then statistically analyzed by using the Social Package for Statistical System (SPSS) version 21.0 (IBM Corp., Armonk, NY, USA) with paired t-test. A p-value lesser than 0.05 was considered significant. The Mann-Whitney U test was used to determine the difference between the baseline and three months in both groups.

## Results

All study participants were of the male gender and had a mean age of 39.75 ± 7.25 years. No statistically significant differences were observed between the mean age of the two groups (p=0.584).

Among the selected patients, two had to be dropped from the study due to distortions in radiographs while four had to be withdrawn from this study as they could not participate in the follow-up appointments. Hence, a total of 23 patients had implant placement following flap reflection while 21 had implant insertion without raising a flap. A loss of 0.08 mm was observed in the flap group while 0.03 mm was seen in the flapless group on the mesial aspect. On the other hand, losses of 0.35 mm and 0.2 mm were observed on the distal aspects of both groups, respectively. As shown in Table 1, a statistical significance between both groups was found in terms of loss of alveolar crestal bone. 

**Table 1:** Table showing loss of crestal bone height in both the study groups

Measurement criteria for alveolar crestal bone loss			
Time duration	Implant placement with flap surgery (Group 1)	Implant placement with flapless surgery (Group 2)	p-value
0 to 3 months	0.05 mm (mesial)	0.02 mm (mesial)	0.04
0 to 6 months	0.08 mm (mesial)	0.03 mm (mesial)	
0 to 3 months	0.21 mm (distal)	0.1 mm (distal)	0.05
0 to 6 months	0.35 mm (distal)	0.2 mm (distal)	

## Discussion

The usually followed standardized clinical protocol for insertion of dental implants is a ‘two-staged’ flap-based approach that involves reflection of soft tissue flap. This surgical protocol has demonstrated excellent results in the long term. The main drawback of using flap surgery for implant placement is the considerable loss of alveolar crestal bone [2].

Various factors may be affecting alveolar crestal bone which are also highly variable in each individual. These factors may include- the design of an implant, the technique used for surgery, the design of a prosthesis along with loading factors [5]. The success of osseointegration of the implant depends on micro and macrotopography. The topography of dental implants can be broken down into macro, micro, and nanoscales. The visible geometry (mm scale) of an implant serves as the basis for its macrotopography. A suitable macrogeometry is linked to a suitable drill hole preparation for dental implants. On a micrometer scale, microtopography and microroughness are related. To improve osseointegration, a variety of surface modifications have been applied to implants using subtractive and additive techniques, including physical (turning, blasting), chemical (acid etching, alkali), electrochemical (electropolishing anodizing), deposition (plasma-spraying, sol-gel), and biochemical (proteins). Surface implants can be made with nanosized hydroxyapatite or titanium dioxide (TiO2) particles to create nanostructures [6].

In the traditional (flap surgery) procedure, the implant site is prepared by exposing the bony ridge with the reflection of the flap and this exposes the underlying. This is associated with postoperative swelling and considerable pain to the patient. Flapless implant surgery is considered an advanced procedure and here, an implant is placed without raising a gingival flap. The crestal bone area is considered a significant indicator of implant health especially as it bears the maximum stress around an implant. It has been found that elevation of the mucoperiosteal flap leads to bone resorption due to a decrease in periosteal blood supply [7-9]. However, there is insufficient evidence in the literature comparing crestal bone height in both surgical techniques. Both the amount as well as the quality of supporting bone are critical for osseointegration.

The null hypothesis was accepted in the present study since flapless implant placement had lesser crestal bone loss compared to the flap technique. This is mainly because the crestal bone area which determines the implant health mainly depends on the periosteal blood supply. And in the flapless technique, bone vascularization is preserved. Once the periosteum is stripped, there is a definite loss of blood supply to the crestal bone leading to increased bone loss which is found in the flap technique [9,10].

Our study findings are supported by Gupta et al. who similarly observed a greater reduction in crestal bone height in implants placed when the mucoperiosteal flap was raised [11]. The flapless surgical technique is performed by either reaching the alveolar bone by punching out a minor amount of soft tissue that may be required for preparing an osteotomy site or performing the osteotomy procedure directly via overlying soft tissue [12]. It has been suggested that the surgical technique without raising a mucoperiosteal flap helps in minimizing inter-proximal loss of crestal bone [13-17]. 

Marginal bone loss is determined by measuring the interproximal height of bone which is defined as the distance between the apical ends of the first thread of the implant and the most coronal point of the interproximal crestal bone [9]. In a flapless technique, the intact blood supplies from soft tissue assist in the maintenance of nutrition, which is a critical factor in preventing initial bone loss around an implant. This results in the maintenance of soft tissue contours mainly for esthetics due to better quality of osseointegration [12-14].

In the present study, an early-stage loading was followed after single-stage implant placement by traditional flap, and flapless surgery. Early loading has been extensively used in implant therapies, mainly in mandibles with good bone quality. Early implant loading can be defined as the functional loading of implants following a period of healing between six weeks to two months [17,18].

Advantages of flapless implant surgery include: preservation of blood circulation, soft tissue architecture, hard tissue volume, decreased surgical time and accelerated recuperation, minimized bleeding, shorter surgical time, reduced pain, and resumption of normal oral hygiene procedures immediately after the procedure [9].

While the traditional flap surgery does have distinct advantages associated with the procedure, there are several drawbacks such as the surgeon’s inability to visualize anatomical landmarks along with vital anatomical structures and also, an increase in the risk of malposition of angulation or depth of placement of an implant [13,19,20].

Although using the flapless technique remains a blinded procedure, accuracy in implant placement along with angulations may be achieved with good knowledge of the general anatomy of a particular site, thorough diagnostic protocols, and good use of surgical techniques [4]. On average, for an accurate assessment of various merits associated with the flapless technique, large numbers of studies with identical functional loading protocols can be used to compare conventional surgical intervention with flapless implant placement [21,22].

Current study results show that the flapless technique followed by an early functional loading may be a better option for placing implants and is accompanied by reduced resorption of crestal bone compared to the traditional placement of implant using the traditional flap technique followed by an early implant loading.

Limitations

Despite the various benefits of flapless implant surgery, there are chances of damaging neighboring structures such as adjacent teeth roots, buccal cortical bone, important nerves, or the sinus. Other limitations include the surgeon’s lack of ability to imagine anatomic landmarks and vital structures, and the higher chance of risk during implant placement depth. The present study was done on smaller samples. Further studies are needed to validate the result on a larger sample size.

## Conclusions

Various factors may affect alveolar crestal bone such as the design of an implant, technique used for surgery, and design of prosthesis along with loading factors. This study's findings noted a decrease in the height of crestal bone in both techniques which may be accompanied by early loading. However, there was a demonstrated statistical significance associated with less crestal bone height reduction in the flapless technique compared to the conservative flap surgery. 
